# Pyogenic Liver Abscess as Endemic Disease, Taiwan

**DOI:** 10.3201/eid1410.071254

**Published:** 2008-10

**Authors:** Feng-Chiao Tsai, Yu-Tsung Huang, Luan-Yin Chang, Jin-Town Wang

**Affiliations:** National Taiwan University Hospital, Taipei, Taiwan; National Taiwan University, College of Medicine, Taipei

**Keywords:** pyogenic liver abscess, Klebsiella pneumoniae, diabetes mellitus, renal disease, malignancy, epidemiology, research

## Abstract

Pyogenic liver abscess has become a health problem in Taiwanese society. However, the extent of this problem has remained unclear because of the lack of a population-based study. We therefore performed a nationwide analysis of pyogenic liver abscess in Taiwan from 1996 through 2004. We analyzed 29,703 cases from the Taiwan National Health Insurance database and 506 cases from National Taiwan University Hospital. Our analysis showed that the annual incidence of pyogenic liver abscess increased steadily from 11.15/100,000 population in 1996 to 17.59/100,000 in 2004. Diabetes, malignancy, renal disease, and pneumonia were associated with a higher risk for the disease. By contrast, death due to pyogenic liver abscess decreased over time, although population-based abscess-related death increased slightly. Renal disease, malignancy, pneumonia, and heart disease correlated with higher death rates; *Klebsiella* *pneumoniae* infection and therapeutic procedures were related to lower death rates. Diabetes did not significantly change death rates for the 506 patients from the hospital.

## Abstract 2

Increasing incidence and microbiologic shift might have changed the manifestation of this condition.

The epidemiology of pyogenic liver abscess has changed dramatically in recent years (*1*). Previously, although incidence was considered rare, the condition was associated with high illness and death rates, usually due to underlying hepatobiliary diseases and polymicrobial infection (*2*), with *Escherichia coli* as the major pathogen (*3*). More recently, investigations in Taiwan suggest the role of cryptogenic or primary liver abscess, i.e., abscesses without underlying hepatobiliary diseases, in pyogenic liver abscess (*4**,**5*). These reports also indicate that diabetes is the major predisposing factor of liver abscess and that *Klebsiella* *pneumoniae* is the primary pathogen. However, these results were obtained from small-scale hospital-based surveys, which could not provide a panoramic view of the disease. To confirm these observation-based results, we conducted a large-scale, unbiased investigation.

In addition to epidemiology, the pathogenesis of liver abscess caused by *Klebsiella* spp. has also been extensively studied, but the mechanism is still not clear. MagA, an outer-membrane protein contributing to capsular polysaccharide formation, coexists with serotype K1 and has been identified as the major virulence factor of *K.* *pneumoniae* (*6*). MagA-positive (or serotype K1) *K.* *pneumoniae* is accordingly recognized as the main pathogen of pyogenic liver abscess (*7**,**8*). Nevertheless, how diabetes increases the risk for *Klebsiella* spp. liver abscess is still not clear. Further research is needed on whether pyogenic liver abscess is affected by immunocompromised conditions, such as malignancy, renal failure, postorgan transplantation, or HIV infection.

To clarify the epidemiology and pathogenesis of pyogenic liver abscess, we used information gathered by the Taiwan National Health Insurance (NHI) program, which was initiated in 1995 by the government to cover most Taiwanese citizens. In 2005, 91.25% of healthcare providers were enrolled in the program and 99% of Taiwanese were insured (*9*). Consequently, since 1995, the program has obtained comprehensive health data on the population in Taiwan. In this study, we used NHI data to study the incidence and death rates caused by pyogenic liver abscess in Taiwan and to investigate factors modifying the manifestations of this disease.

## Methods

### Patients

We requested data on patients with pyogenic liver abscess from the Taiwan NHI program. Cases were selected by using the following criteria: patients were hospitalized and reported before 2004, and the discharge diagnoses included abscess of liver per the International Classification of Diseases, 9th revision, Clinical Modication (ICD-9-CM 572.0) but excluded amebic liver abscess (ICD-9-CM 006.3). Though we selected cases documented up to the end of 2004, the database could not provide information from patients who had not yet been discharged. Those admitted before December 31, 2004, but discharged during or after 2005 were therefore not included in our database. This exclusion results in the underestimation of case-patients admitted at the end of 2004.

Data on 29,965 case-patients were collected. After excluding patients discharged before 1996 and those without clear records regarding age or sex, we enrolled 29,703 case-patients in our study. Patient data were anonymous. Names of these patients were not included, and patient and healthcare provider identification numbers were encrypted.

This primary set of data included the date of admission and discharge, age, sex, diagnoses (up to 5), procedures (up to 5), outcome at discharge (recovered or died), and the fees charged to patients. Laboratory data, including microbiologic data and medication, were not included. Any underlying diseases were determined by the diagnoses listed in the medical records, which were coded by ICD-9-CM.

Because *K*. *pneumoniae* is the major pathogen of primary pyogenic liver abscess in Taiwan, it is expected to play an important role in the pathogenesis and prognosis of this disease. Unfortunately, the NHI database does not include microbiologic data. To compensate for this, we reviewed the records of patients in National Taiwan University Hospital (NTUH). This hospital is a public medical center in Taipei, functioning both as a primary care hospital and as a tertiary referral center (*10*). As the leading hospital in Taiwan (*10*) with a 113-year history (*11*), NTUH serves patients and accepts referrals evenly distributed from every part of Taiwan. The hospital provides care for ≈2,000 inpatients and 7,000 outpatients a day (*11*), which are ≈3.5% and 2%, respectively, of persons included in the NHI database (*12*). Therefore, the patients of NTUH are representative of all of the patients in Taiwan, without substantial bias but may be skewed slightly to the severe side. We selected case-patients from this hospital using the same criteria mentioned above, except that the discharge year was between January 1, 2000, and December 31, 2004; complete microbiologic data was preserved in the NTUH laboratory only after 2000. These patients were included in the NHI database anonymously. For case-patients from NTUH, we reviewed actual medical records and obtained microbiologic data from the hospital’s laboratory.

### Statistical Analysis

Numerical data were compared by Student *t* test or paired *t* test. Categorical data were processed by χ^2^ test. Pearson correlation coefficients and χ^2^ goodness-of-fit test were used to estimate the trend of incidence and death over time. Unfortunately, incidence and death from different years could not be directly compared because the population structure changed slightly over the study period. To correct the bias, we calculated age-standardized incidence and death rates. The correction was based on age-specific population data in 1996. Finally, risk factor analysis was conducted by using the binary logistic regression and curve estimation methods by SPSS version 11.0 for Macintosh (SPSS, Inc. Chicago, IL, USA).

## Results

### Demographic Data

A total of 29,703 case-patients from the NHI database were enrolled in our analysis (Table 1). Ages of these patients ranged from <1 through 106 years of age, with a median age of 61 years; a total of 9,904 (33.3%) had diabetes mellitus, 3,079 (10.4%) had cirrhosis of the liver, 4,350 (14.6%) had cholelithiasis, and 4,115 (13.9%) had concomitant malignancy.

**Table 1:** Demographic data from National Health Insurance database, Taiwan, 1996–2004

Item	Female	Male	Total	p value*
Sex, no. (%)	11,377 (38.3)	18,326 (61.7)	29,703 (100)	
Age: range (median), y	0–106 (63.90)	0–101 (58.98)	0–106 (61.00)	<0.001†
Age: mean (SD), y	62.13 (14.87)	57.58 (16.03)	59.32 (15.75)	<0.001‡
Hospitalization: mean (SD), d	18.00 (13.80)	16.91 (12.74)	17.33 (13.17)	<0.001‡
Diabetes mellitus, no. (%)	3,998 (35.1)	5,906 (32.2)	9,904 (33.3)	<0.001§
Cirrhosis, no. (%)	854 (7.5)	2,225 (12.1)	3,079 (10.4)	<0.001§
Renal disease, no. (%)	772 (6.8)	1,191 (6.5)	1,963 (6.6)	0.334§
Hypertension, no. (%)	1,491 (13.1)	1,866 (10.2)	3,357 (11.3)	<0.001§
Heart disease, no. (%)	826 (7.3)	1,118 (6.1)	1,944 (6.5)	<0.001§
Cerebrovascular accident, no. (%)	364 (3.2)	454 (2.5)	818 (2.8)	<0.001§
Cholelithiasis, no. (%)	2,121 (18.6)	2,229 (12.2)	4,350 (14.6)	<0.001§
Hepatobiliary malignancy, no. (%)	863 (7.6)	1,769 (9.7)	2,632 (8.9)	<0.001§
Other malignancy, no. (%)	546 (4.8)	937 (5.1)	1,483 (5.0)	0.227§
Pneumonia, no. (%)	675 (5.9)	1,273 (6.9)	1,948 (6.6)	0.001§
Urinary tract infection, no. (%)	923 (8.1)	697 (3.8)	1,620 (5.5)	<0.001§
Acute viral hepatitis, no. (%)	228 (2.0)	657 (3.6)	885 (3.0)	<0.001§
Peptic ulcer, no. (%)	985 (8.7)	1,623 (8.9)	2,608 (8.8)	0.557§
Abscess drainage no. (%)	3,886 (34.2)	6,082 (33.2)	9,968 (33.6)	0.082§
Biliary procedure no. (%)	1,967 (17.3)	2,361 (12.9)	4,328 (14.6)	<0.001§
Deaths, no. (%)	1,286 (11.3)	1,954 (10.7)	3,240 (10.9)	0.085§

*p<0.05 is considered statistically significant. †Mann-Whitney U test. ‡Student *t* test. §χ^2^ test.

Average hospitalization was 17.33 days. The proportions of patients who received abscess drainage and biliary procedures (endoscopic or surgical biliary drainage) were 33.6% and 14.6%, respectively. The death rate was 10.9%.

Male patients dominated the sample population (18,326/29,703, 61.7%) and, on average, were 5 years younger than their female counterparts (57.58 ± 16.03 vs. 62.13 ± 14.87 years, p*<*0.001). Besides the difference in age, more female patients had concomitant cholelithiasis (18.6% vs. 12.2%, p*<*0.001). Female patients also received biliary procedures more often (17.3% vs. 12.9%, p*<*0.001) than did male patients.

**Figure 1:**
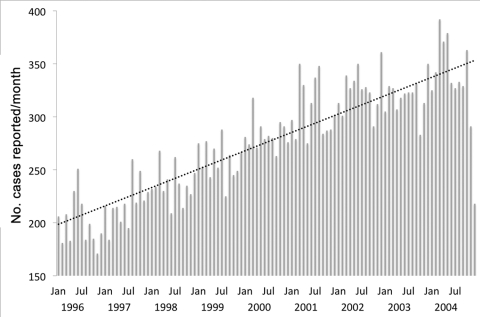
Number of new reported cases of pyogenic liver abscess recorded monthly in the National Health Insurance database, Taiwan, January 1996 through December 2004.

We then investigated the data over time. An average of 3,300 cases/year or 275 cases/month were reported. The number of reported cases increased from 2,400 in 1996 to 3,991 in 2004 (Table 2), with a stable increase rate of 1.44 more new cases per month (Figure 1); the decline in cases at the end of 2004 is possibly due to the incomplete recruitment of cases as described in the Methods section.

**Table 2:** Incidence rates and deaths from pyogenic liver abscess, National Health Insurance database, Taiwan, 1996–2004*

Year	Case-patients with pyogenic liver abscess				Deaths from pyogenic liver abscess		
	Female	Male	Total		Female	Male	Total
1996	945 (9.03)	1455 (13.15)	2400 (11.15)		112 (1.07)	184 (1.66)	296 (1.38)
1997	1045 (9.88)	1562 (13.99)	2607 (11.99)		135 (1.28)	173 (1.55)	308 (1.42)
1998	1130 (10.58)	1701 (15.13)	2831 (12.91)		151 (1.41)	189 (1.68)	340 (1.55)
1999	1162 (10.78)	1936 (17.11)	3098 (14.02)		138 (1.28)	213 (1.88)	351 (1.59)
2000	1300 (11.94)	2094 (18.38)	3394 (15.24)		147 (1.35)	201 (1.76)	348 (1.56)
2001	1453 (13.25)	2218 (19.39)	3671 (16.38)		160 (1.46)	258 (2.25)	418 (1.87)
2002	1406 (12.74)	2485 (21.64)	3891 (17.28)		151 (1.37)	259 (2.26)	410 (1.82)
2003	1434 (12.93)	2386 (20.72)	3820 (16.90)		143 (1.29)	238 (2.07)	381 (1.69)
2004	1502 (13.47)	2489 (21.57)	3991 (17.59)		149 (1.34)	239 (2.07)	388 (1.71)
p value†	<0.001	<0.001	<0.001		0.224	<0.001	<0.001

*Data are presented as number (rate per 100,000 population). †χ^2^ goodness-of-fit test. p<0.05 is considered statistically significant.

The annual number of case-patients increased with age; peak incidence for women was seen in those 60–64 years of age and in men 65–69 years of age. A slight decline was noted in case-patients 55–59 years of age, a reflection of decreased birth rates during World War II. Men <85 years of age had more liver abscess than women but the opposite result was seen in women >85 years of age.

### Incidence and Risk Factors

**Figure 2:**
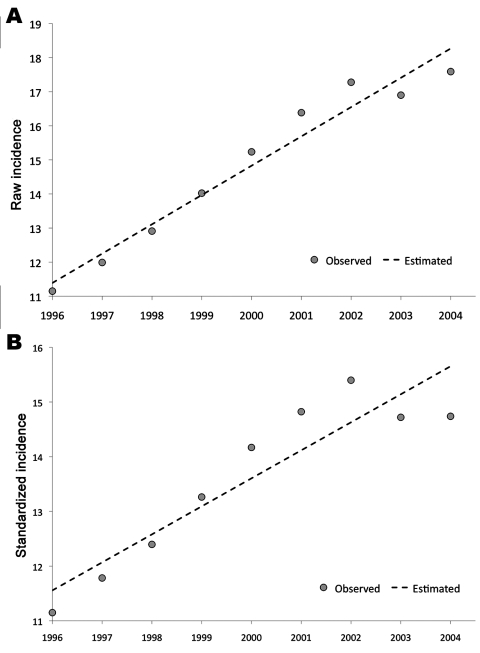
Incidence of pyogenic liver abscess in Taiwan, showing a steady increase from 1996 to 2004. The incidence is expressed as the number of new cases reported from the population (the National Health Insurance database) per year. A) The linear increase of the primary incidence data (raw incidence) with the year can be described by this formula: incidence (×1/100,000) = 0.860 × year – 1704.66 (*r* = 0.978, p<0.001). B) The linear increase of the standardized incidence data (the incidence normalized according to the age distribution of the population) with the year can be described by this formula: incidence (×1/100,000) = 0.512 × year – 1010.68 (*r* = 0.923, p<0.001). *r*, Pearson correlation coefficient.

The gross incidence of pyogenic liver abscess from 1996 through 2004 was 14.87 cases/100,000 population/year (17.94 male cases/100,000 population and 11.65 female cases/100,000 population). The annual increase of incidence was 0.86 cases/100,000 population (*r =* 0.98, p*<*0.001) (Figure 2, panel **A**). When we calculated age-standardized incidences, the increase rate was 0.51 cases/100,000 population/year (*r =* 0.92, p*<*0.001) (Figure 2, panel **B**). The age of highest incidence in men was in those 80–84 years of age (86.71 cases/100,000 population); for women, the highest incidence was in those 85–89 years of age (79.80 cases/100,000 population).

Several factors were associated with the increased incidence of liver abscess. Because incidence data from each year could not be merged directly, we chose the cases reported in 2004 to evaluate the relative risk for each factor (Table 3). Diabetes mellitus and malignancy were associated with a ≈10-fold increased risk, while renal disease and pneumonia tripled and quadrupled the incidence of liver abscess, respectively. We did not investigate the interactions among these factors because it required detailed health records of each person in the Taiwan population, which would violate patient confidentiality regulations.

**Table 3:** Factors associated with increased incidence in pyogenic liver abscess based on cases reported in the National Health Insurance database, Taiwan, 2004*

Factor	Incidence with factor	Incidence without factor	Relative risk (95% CI)
Sex (M)	21,565	13,474	1.601 (1.501–1.707)
Acute viral hepatitis	50,798	17,090	2.973 (2.511–3.466)
Diabetes mellitus	111,998	12,322	9.098 (8.520–9.716)
Malignancy	162,610	15,176	10.731 (9.840–11.702)
Hypertension	23,989	16,772	1.430 (1.313–1.559)
CVA	22,756	17,471	1.303 (1.083–1.567)
Pneumonia	77,048	16,541	4.661 (4.145–5.240)
Renal disease	62,795	16,878	3.722 (3.250–4.263)
Heart disease	19,494	17,468	1.116 (0.986–1.264)

*Incidence is expressed as cases/100,000 population. CI, confidence interval; CVA, cerebrovascular accident.

### Change in Death Rates

**Figure 3:**
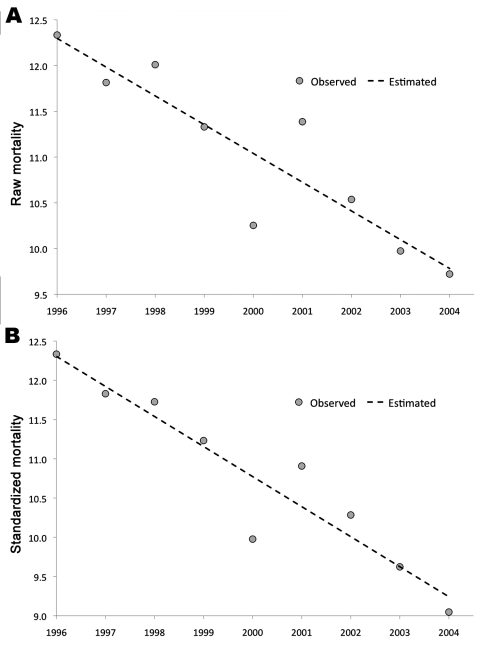
Deaths from pyogenic liver abscess in Taiwan, showing a steady decrease from 1996 to 2004. Mortality rate is expressed as the number of deaths reported from pyogenic liver abscess cases per year. A) The linear decrease of the primary mortality data (raw mortality) with the year can be described with this formula: mortality (×1/100) = −0.314 × year + 639.58 (*r* = 0.910, p<0.001). B) The linear decrease of the standardized mortality data (the mortality normalized according to the age distribution of the population) with the year can be described with this formula: mortality (×1/100) = −0.383 × year +776.59 (*r* = 0.944, p<0.001). *r*, Pearson correlation coefficient. p<0.05 is considered statistically significant.

In contrast to increased incidence rates from 1996 through 2004, the disease-specific death rate declined steadily from 12.33% in 1996 to 9.72% in 2004 while the number of deaths caused by pyogenic liver abscess increased slightly over this same timeframe (Figure 2, panel **B**). The yearly change in the death rate was –0.31% (*r =* 0.91, p*<*0.001) (Figure 3, panel **A**). When we calculated age-standardized death rates, the decrease was 0.38%/year (*r =* 0.94, p*<*0.001) (Figure 3, panel **B**). Death rates did not differ much between males and females (10.7% vs. 11.3%) (Table 1). Death rates increased slowly for men and women 35–85 years of age and peaked for those 90–94 years of age (33.81%). Minor peaks were noted for adolescents 10–14 years of age (19.35%) and for young men 20–24 years of age (12.36%), respectively.

We tested case-patients with binary logistic regression to identify the factors contributing to increased death rates (Table 4). Multivariate analysis showed an increased risk of 1.7%/year of age. Renal disease and malignancy were respectively associated with a 2.5- and a 2-fold increased risk, followed by pneumonia (1.5-fold) and heart disease (1.3-fold). Some underlying diseases were associated with a lower risk for death, including diabetes (0.85-fold), peptic ulcer (0.75- fold), urinary tract infection (0.74-fold), hypertension (0.44-fold), and cholelithiasis (0.69-fold). Therapeutic procedures were also related to the lower risk of death (abscess drainage 0.57-fold, biliary drainage 0.73-fold), compatible with our expectations.

**Table 4:** Binary logistic regression analysis showing factors associated with death from pyogenic liver abscess, Taiwan, 1996–2004*

Factor	Univariate analysis				Multivariate analysis		
	Odds ratio	p value†	95% CI		Odds ratio	p value†	95% CI
Age, y	1.018	<0.001	1.015–1.020		1.017	<0.001	1.015–1.020
Sex (M)	0.937	0.085	0.869–1.009		0.930	0.067	0.861–1.005
Diabetes mellitus	0.813	<0.001	0.750–0.880		0.850	<0.001	0.782–0.925
Abscess drainage	0.541	<0.001	0.497–0.590		0.571	<0.001	0.523–0.624
Biliary procedure	0.635	<0.001	0.564–0.715		0.728	<0.001	0.640–0.828
Peptic ulcer	0.778	<0.001	0.676–0.895		0.751	<0.001	0.651–0.867
Urinary tract infection	0.851	0.063	0.718–1.009		0.735	0.001	0.617–0.875
Cirrhosis	1.027	0.663	0.912–1.156		–	–	–
Renal disease	2.599	<0.001	2.322–2.909		2.452	<0.001	2.181–2.756
Hypertension	0.480	<0.001	0.414–0.557		0.435	<0.001	0.374–0.506
Cerebrovascular accident	1.405	0.001	1.153–1.713		1.219	0.058	0.993–1.497
Cholelithiasis	0.599	<0.001	0.530–0.676		0.693	<0.001	0.608–0.790
Hepatobiliary malignancy	1.745	<0.001	1.564–1.947		1.850	<0.001	1.646–2.080
Other malignancy	2.151	<0.001	1.883–2.456		2.136	<0.001	1.859–2.454
Pneumonia	1.770	<0.001	1.563–2.003		1.516	<0.001	1.333–1.723
Acute viral hepatitis	0.481	<0.001	0.361–0.642		0.431	<0.001	0.321–0.578
Heart disease	1.384	<0.001	1.212–1.581		1.254	0.001	1.092–1.440

*CI, confidence interval. †p<0.05 is considered statistically significant.

### *K. pneumoniae* and Pyogenic Liver Abscess at NTUH

To compensate for the deficiency of microbiologic data in the NHI database, we reviewed the medical records of case-patients with primary pyogenic liver abscess admitted to NTUH from 2000 through 2004. In total, 506 case-patients were enrolled, ≈3.70% of all case-patients in Taiwan (13,672) during the same period. This ratio is similar to the general NTUH: Taiwan inpatient ratio (3.50%) (*12*), suggesting that liver-abscess cases in NTUH are probably a microcosm for the liver-abscess patients in all of Taiwan. Demographic data are shown in Table 5. Compared with the NHI database, case-patients in NTUH had longer hospitalization periods and higher frequencies of heart disease and malignancy, implying severe underlying conditions. However, the ratios of sepsis, meningitis, endophthalmitis, and pneumonia were similar between NTUH and Taiwan data, so the severity of primary liver abscess in NTUH patients and Taiwan as a whole were equal. Besides, NTUH case-patients were associated with higher rates of abscess drainage and lower death rates, indicating that the quality of medical care for those patients is probably better than the average level of medical care provided in Taiwan.

**Table 5:** Demographic data on patients with primary pyogenic liver abscess from NTUH and NHI databases, Taiwan, 2000–2004*

Item	NHI database	NTUH	p value†
Total no. case-patients	13,672	506 (3.70)	
Age, y, range (median)	0–100 (59.95)	0–90 (57.50)	
Age, y, mean (SD)	58.31 (16.37)	55.92 (16.93)	0.001
Hospitalization, mean (SD)	17.32 (12.80)	30.13 (34.08)	<0.001
M/F, no. (%)	8,652/5,020 (63.3/36.7)	329/177 (65.0/35.0)	0.426
Diabetes mellitus, no. (%)	5,428 (39.7)	140 (27.7)	<0.001
Cirrhosis, no. (%)	1,153 (8.4)	19 (3.8)	<0.001
Renal disease, no. (%)	1,113 (8.1)	47 (9.3)	0.355
Hypertension, no. (%)	1,947 (14.2)	88 (17.4)	0.047
Heart disease, no. (%)	1,038 (7.6)	53 (10.5)	0.017
Cerebrovascular accident, no. (%)	461 (3.4)	17 (3.4)	0.988
Other malignancy, no. (%)	930 (6.8)	77 (15.2)	<0.001
Sepsis, no. (%)	3,252 (23.8)	137 (27.1)	0.089
Meningitis, no. (%)	155 (1.1)	6 (1.2)	1.000
Endophthalmitis, no. (%)	226 (1.7)	10 (2.0)	0.593
Pneumonia, no. (%)	1,141 (8.3)	54 (10.7)	0.064
Urinary tract infection, no. (%)	916 (6.7)	20 (4.0)	0.015
Acute viral hepatitis, no. (%)	400 (2.9)	10 (2.0)	0.211
Peptic ulcer, no. (%)	1,161 (8.5)	20 (4.0)	<0.001
Abscess drainage, no. (%)	4,795 (35.1)	292 (57.7)	<0.001
Biliary procedure, no. (%)	790 (5.8)	15 (3.0)	0.007
Deaths, no. (%)	1,410 (10.3)	31 (6.1)	0.002

*Data expressions and statistical methods in this table are the same as in Table 1. NTUH, National Taiwan University Hospital; NHI, National Health Insurance. †p<0.05 is considered statistically significant.

Among the 506 cases, 358 had positive culture results (Table 6), and 286 (79.9%) of 358 case-patients showed *K*. *pneumoniae* infection. Patients with *Klebsiella* spp. infection had a lower death rate (2.4% vs. 11.1%; p *=* 0.004), less mixed bacterial infection (4.5% vs. 26.4%; p*<*0.001), and less underlying malignancy (5.2% vs. 20.8%; p*<*0.001). Of case-patients with *Klebsiella* spp. liver abscess, 35% were associated with diabetes mellitus. The prevalence of diabetes in case-patients with other micromicrobial infections was 18.1% (p *=* 0.007). Binary logistic regression analysis showed that *Klebsiella* spp. infection was associated with decreased death rates (relative risk 0.20, p *=* 0.003); the role of diabetes was neutral (relative risk 1.09, p *=* 0.88) (Table 7). Therefore, the low death rates in case-patients with diabetes who also had liver abscess were probably attributed to *Klebsiella* spp. infection.

**Table 6:** *Klebsiella* spp. liver abscess compared with other primary pyogenic liver abscess, NTUH, Taiwan, 1996–2004*

Item	*Klebsiella* spp.	Non-*Klebsiella* spp.	p value†
Total no.	286	72	
Male gender, no. (%)	191 (66.8)	45 (62.5)	0.490
Deaths, no. (%)	7 (2.4)	8 (11.1)	0.004
Abscess drainage, no. (%)	204 (71.3)	49 (68.1)	0.664
Biliary procedure, no. (%)	2 (0.7)	9 (12.5)	<0.001
Mixed infection, no. (%)	13 (4.5)	19 (26.4)	<0.001
Diabetes mellitus, no. (%)	100 (35.0)	13 (18.1)	0.007
Peptic ulcer, no. (%)	10 (3.5)	7 (9.7)	0.055
Urinary tract infectio, no. (%)	14 (4.9)	2 (2.8)	0.749
Renal disease, no. (%)	25 (8.7)	6 (8.3)	1.000
Hypertension, no. (%)	55 (19.2)	14 (19.4)	1.000
Heart disease, no. (%)	28 (9.8)	9 (12.5)	0.517
Cerebrovascular accident, no. (%)	7 (2.4)	3 (4.2)	0.427
Malignancy, no. (%)	15 (5.2)	15 (20.8)	<0.001
Cirrhosis, no. (%)	5 (1.7)	2 (2.8)	0.632
Pneumonia, no. (%)	35 (12.2)	6 (8.3)	0.414
Viral hepatitis, no. (%)	4 (1.4)	1 (1.4)	1.000
Meningitis, no. (%)	3 (1.0)	0 (0.0)	0.613
Endophthalmitis	9 (3.1)	0 (0.0)	0.214
Age, y, mean (SD)	57.39 (15.52)	55.25 (19.26)	0.384
No. hospitalizations, mean (SD)	26.12 (16.36)	43.06 (69.64)	0.044

*NTUH, National Taiwan University Hospital. The χ^2^ test and Student *t* test were used for statistical analyses. †p<0.05 is considered statistically significant.

**Table 7:** Factors modifying the death rates from primary pyogenic liver abscess analyzed by binary logistic regression, NTUH, Taiwan, 2000–2004*

Factor	Univariate analysis				Multivariate analysis		
	Odds ratio	p value†	95% CI		Odds ratio	p value†	95% CI
Age	1.034	0.074	0.997–1.073		–	–	–
Gender	0.436	0.117	0.154–1.231		–	–	–
*Klebsiella*	0.201	0.003	0.070–0.574		0.223	0.009	0.072–0.690
Diabetes mellitus	1.088	0.880	0.363–3.260		–	–	–
Renal disease	4.256	0.019	1.269–14.271		6.172	0.006	1.665–22.884
Heart disease	1.354	0.698	0.293–6.247		–	–	–
Malignancy	4.433	0.016	1.319–14.900		2.922	0.119	0.759–11.254
Cirrhosis	4.012	0.212	0.452–35.613		–	–	–
Abscess drainage	0.345	0.045	0.122–0.977		0.351	0.063	0.116–1.060
Hospitalization days	1.002	0.793	0.990–1.013		–	–	–

*NTUH, National Taiwan University Hospital; CI, confidence interval. †p<0.05 is considered statistically significant.

## Discussion

We present a nationwide population-based report of pyogenic liver abscess in Taiwan. Taiwan started the NHI program in 1995, with a coverage rate of ≈99% of its population. By providing national healthcare, the system collected medical records from virtually every person seeking medical help in Taiwan so many nationwide surveys were automatically completed. The database, therefore, reflects a complete and unbiased picture of general health conditions in Taiwan.

Our data suggests Taiwan is endemic for pyogenic liver abscess. This disease had been considered rare in the past; previous reports showed an annual incidence rate of 2.3/100,000 (*13*) and 1.0/100,000 (*14*) in Canada and Denmark, respectively. In our report, however, the incidence rate was 10× higher (11.99/100,000 in 1996 and up to 17.59/100,000 in 2004). Such high incidence rates and rapid increase indicate the true growth of this disease because the data could not be explained solely by the change in population structure or by improved detection capacity of medical professionals. First, the diagnostic tools and methods for pyogenic liver abscess did not remarkably change during our study. Furthermore, the incidence did not explode but increased steadily over time. Thus, the increase is unlikely to have been caused by improved diagnostic sensitivity. Second, to eliminate bias induced by the change in population structure over time, we calculated age-standardized incidence. The increased incidence remains significant after correction (Figure 2, panels **A**, **B**). Hence, endemic pyogenic liver abscess possibly existed long before these data were first collected in Taiwan, even though the situation has recently become much worse. During the study period, magA-positive *K*. *pneumoniae* was identified as the major cause of pyogenic liver abscess in Taiwan (*6**,**15*). Although culture data were lacking in the NHI database, 80% of NTUH case-patients had positive culture data that showed *Klebsiella* spp. A recent nationwide report from South Korea also showed that the proportion of *K*. *pneumoniae* infection increased dramatically in that country over time from ≈0% in 1955–1969 up to 78.2% during 2004–2005 (*8*). We can therefore infer that the high incidence of pyogenic liver abscess in Taiwan is related to *Klebsiella* spp. infection.

In contrast to the rise of incidence over time, death rates from pyogenic liver abscess decreased. In our analysis, the death rate from pyogenic liver abscess in Taiwan was 10.9% during 1996–2004 (Table 1) while the inhospital death rate was 6.1% (Table 5). These rates are much lower than those in earlier reports, 25% (*16*) to 50% (*14*), but are consistent with those in more recent reports, in which population-based death rates were ≈10% (*13**,**14*) while inhospital death rates ranged from 6%–8% (*15**,**17**,**18*). This decrease might be multifactorial.

First, the decrease in death rates might be the result of the dramatic increase of case-patients with pyogenic liver abscess. In a previous report, Jepsen et al. suggested that the dramatic decrease in death rates in Denmark between 1977 and 2002 resulted from improved diagnostic tools (*14*). This reason might also apply in Taiwan because the liver abscess–related death rate in the general population actually increased from 1.38/100,000 in 1996 to 1.80/100,000 in 2004. However, the increase was not steady, with a peak of 1.94/100,000 in 2001 followed by a decrease, indicating a true decrease of disease-specific deaths. Thus, diagnostic sensitivity could not explain the whole condition.

Second, the advance of medical care might have contributed to the decrease in mortality rates. Because abscess drainage was reported to improve the outcome (*2**,**15**,**19*) and *Klebsiella* spp. was associated with a relatively benign course under aggressive medical care (*5**,**20*), we hypothesized that the decrease in death rates was due to the increase of *Klebsiella* spp. infection and the increase of abscess drainage. To test this hypothesis, we calculated the trends of death-related factors over time in the NHI database. As indicated in Table 8, the decrease in death rates was chronologically compatible with the increase of abscess drainage (+1.38% per year, *r =* 0.88, p *=* 0.002). Although the database did not include culture data, the annual increase of diabetes (+0.69%, *r =* 0.86, p *=* 0.003) and pneumonia (+0.28%, *r =* 0.90, p *=* 0.001) suggested the concomitant annual increase of *Klebsiella* spp. infection. Therefore, the significant decrease of liver abscess-related deaths in recent years is caused by a microbiologic shift (more *Klebsiella* spp. infections) and better medical care (more abscess drainage).

**Table 8:** Chronological changes of factors associated with pyogenic liver abscess, NHI database, Taiwan, 1996–2004*

Year (no. cases)	Abscess drainage, no. (%)	Biliary procedure, no. (%)	Renal disease, no. (%)	Diabetes mellitus, no. (%)	Hepatobiliary malignancy, no. (%)	Other malignancy, no. (%)	Pneumonia, no. (%)	Heart disease, no. (%)	Deaths, no. (%)
1996 (2,400)	589 (24.5)	316 (13.2)	123 (5.1)	696 (29)	149 (6.2)	81 (3.4)	132 (5.5)	137 (5.7)	296 (12.3)
1997 (2,607)	729 (28)	389 (14.9)	156 (6)	819 (31.4)	210 (8.1)	106 (4.1)	155 (5.9)	154 (5.9)	308 (11.8)
1998 (1,831)	932 (32.9)	473 (16.7)	161 (5.7)	911 (32.2)	276 (9.7)	124 (4.4)	168 (5.9)	186 (6.6)	340 (12)
1999 (3,098)	1,055 (34.1)	480 (15.5)	198 (6.4)	1,005 (32.4)	302 (9.7)	137 (4.4)	179 (5.8)	194 (6.3)	351 (11.3)
2000 (3,394)	1,190 (35.1)	484 (14.3)	258 (7.6)	1,139 (33.6)	292 (8.6)	171 (5)	193 (5.7)	198 (5.8)	348 (10.3)
2001 (3,671)	1,233 (33.6)	515 (14)	286 (7.8)	1,241 (33.8)	340 (9.3)	203 (5.5)	246 (6.7)	254 (6.9)	418 (11.4)
2002 (3,891)	1,355 (34.8)	604 (15.5)	279 (7.2)	1,339 (34.4)	372 (9.6)	206 (5.3)	290 (7.5)	264 (6.8)	410 (10.5)
2003 (3,820)	1,387 (36.3)	519 (13.6)	281 (7.4)	1,411 (36.9)	347 (9.1)	195 (5.1)	282 (7.4)	290 (7.6)	381 (10.0)
2004 (3,991)	1,498 (37.5)	548 (13.7)	221 (5.5)	1,343 (33.7)	344 (8.6)	260 (6.5)	303 (7.6)	267 (6.7)	388 (9.7)
Change per y, %	+1.38	(–0.10)	+0.17	+0.69	+0.20	+0.31	+0.28	+0.17	(–0.31)
*r*	0.88	–0.23	0.46	0.86	0.49	0.93	0.90	0.75	–0.91
p value†	0.002	0.547	0.208	0.003	0.179	<0.001	0.001	0.020	0.001

*NHI, National Health Insurance; *r*, Pearson correlation coefficient. †p<0.05 is considered statistically significant.

Our study suggests that both underlying renal disease and malignancy increased the incidence and mortality rates of pyogenic liver abscess. A previous survey in patients with end-stage renal disease showed the in-hospital prevalence of liver abscess to be 130.59/100,000 and the death rate to be 33.30% (*21*). Patients died of septic shock despite aggressive management in 50% of these cases. In our data, renal disease triples the incidence of pyogenic liver abscess and doubles the death rates, compatible with the previous report. Similarly, underlying malignancy had been well recognized as an important aggravating factor (*13**,**22*) of liver abscess. Our data also showed 10-fold and 2-fold increases in the incidence and mortality rates of pyogenic liver abscess. In addition to the fact that tumors from the hepatobiliary region might mimic abscesses (*23**–**25*), tumors in other regions were also associated with poor prognoses (*26**,**27*), confirming malignancy itself as an independent aggravating factor. Because the prevalence of both renal disease and malignancy has increased in recent years, the threat of pyogenic liver abscess in those patients is becoming more important, worthy of our special notice.

In contrast to renal disease and malignancy, the role of diabetes in mortality rates is controversial. Some reports suggested its association with a more aggressive clinical course (*18**,**28*) while others merely confirmed its coexistence with pyogenic liver abscess (*15**,**19**,**22*). In our analysis of the NHI database, diabetes caused a 9-fold increase of incidence but paradoxically decreased death rates. Further analysis in NTUH case-patients showed that such a decrease was attributed to the high proportion of *Klebsiella* spp. infection and that diabetes played no significant role in prognoses. This result is compatible with a report from a recent population-based case-control study in Denmark which stated that diabetes modified the risk but not the prognosis of pyogenic liver abscess (*29*). Since diabetes compromises host immune systems, its pivotal role in the risk for abscess but minimal role in abscess-related death, indicates a complicated interaction between the pathogens and the immune system of hosts. More in-depth research in this field is necessary.

The NHI database, although almost complete, is limited in 3 aspects. First, it is deficient in microbiologic data. Because the microbiologic data from NTUH is hospital-based, data might not accurately reflect the condition in the general population. Second, because it enables only 5 diagnoses for each case, coding of diagnoses might be biased if the specific case is complicated with >5 underlying diseases. For this reason, some minor conditions, such as peptic ulcer, urinary tract infection, and hypertension, paradoxically decreased death rates in our data. Third, in contrast to the comprehensive data of pyogenic liver abscess, detailed health data for each person in the population are not available. We are therefore unable to estimate the interaction among the risk factors of pyogenic liver abscess in the population (Table 3). Nevertheless, this study still provides a clear picture of pyogenic liver abscess in Taiwan. The rapid and steady increase of cases with pyogenic liver abscess in Taiwan should be noted (Table 2). Although the prognosis of liver abscess patients has improved over time (Figure 3), pyogenic liver abscess-related death in the population continues to increase (Table 2). Furthermore, complex interactions between pyogenic liver abscess, diabetes, renal disease, and malignancy are shown to worsen this condition. Further collaboration among clinical medical practitioners, public health workers, and research scientists is mandatory to fight against such a challenge in the future.
